# Ebola virus disease and critical illness

**DOI:** 10.1186/s13054-016-1325-2

**Published:** 2016-07-29

**Authors:** Aleksandra Leligdowicz, William A. Fischer, Timothy M. Uyeki, Thomas E. Fletcher, Neill K. J. Adhikari, Gina Portella, Francois Lamontagne, Christophe Clement, Shevin T. Jacob, Lewis Rubinson, Abel Vanderschuren, Jan Hajek, Srinivas Murthy, Mauricio Ferri, Ian Crozier, Elhadj Ibrahima, Marie-Claire Lamah, John S. Schieffelin, David Brett-Major, Daniel G. Bausch, Nikki Shindo, Adrienne K. Chan, Tim O’Dempsey, Sharmistha Mishra, Michael Jacobs, Stuart Dickson, G. Marshall Lyon, Robert A. Fowler

**Affiliations:** 1 Interdepartmental Division of Critical Care, University of Toronto, Toronto, ON Canada; 2 Department of Medicine, University of North Carolina, Chapel Hill, NC USA; 3 Centers for Disease Control and Prevention, Atlanta, Georgia USA; 4 Defence Medical Services, Whittington Barracks, Lichfield, UK; 5 Liverpool School of Tropical Medicine, Liverpool, Merseyside UK; 6 Department of Critical Care Medicine, Sunnybrook Health Sciences Centre, Toronto, ON Canada; 7 Emergency NGO, Milan, Italy; 8 Department of Medicine, Université de Sherbrooke, Sherbrooke, Quebec Canada; 9 Polyclinique Bordeaux Nord Aquitaine, Bordeaux, France; 10 Department of Medicine, University of Washington, Seattle, Washington USA; 11 Department of Medicine, University of Maryland, Baltimore, MD USA; 12 Centre de recherche de l’institut Universitaire de Cardiologie et de Pneumologie de Québec, Quebec City, Quebec Canada; 13 Division of Infectious Diseases, University of British Columbia, Vancouver, BC Canada; 14 Department of Paediatrics, University of British Columbia, Vancouver, BC Canada; 15 Infectious Diseases Institute, College of Health Sciences, Makerere University, Kampala, Uganda; 16 Department of Infectious and Parasitic Diseases, Donka Hospital, Conakry, Guinea; 17 Department of Pediatrics, School of Medicine and School of Public Health and Tropical Medicine, Tulane University, New Orleans, LA USA; 18 Department of Preventive Medicine and Biometrics, Uniformed Services University, Bethesda, MD USA; 19 Department of Pandemic and Epidemic Diseases, World Health Organization, Geneva, Switzerland; 20 Division of Infectious Diseases, Sunnybrook Health Sciences Centre, Toronto, ON Canada; 21 Department of Clinical Sciences, Liverpool School of Tropical Medicine, Liverpool, UK; 22 Department of Medicine, University of Toronto, Toronto, ON Canada; 23 Department of Infection, Royal Free London NHS Foundation Trust, London, UK; 24 Acute Medicine and Intensive Care, Derriford Hospital, Plymouth, UK; 25 Department of Infectious Diseases, Emory University Hospital, Atlanta, Georgia USA

**Keywords:** Ebola, Critical care

## Abstract

As of 20 May 2016 there have been 28,646 cases and 11,323 deaths resulting from the West African Ebola virus disease (EVD) outbreak reported to the World Health Organization. There continue to be sporadic flare-ups of EVD cases in West Africa.

EVD presentation is nonspecific and characterized initially by onset of fatigue, myalgias, arthralgias, headache, and fever; this is followed several days later by anorexia, nausea, vomiting, diarrhea, and abdominal pain. Anorexia and gastrointestinal losses lead to dehydration, electrolyte abnormalities, and metabolic acidosis, and, in some patients, acute kidney injury. Hypoxia and ventilation failure occurs most often with severe illness and may be exacerbated by substantial fluid requirements for intravascular volume repletion and some degree of systemic capillary leak. Although minor bleeding manifestations are common, hypovolemic and septic shock complicated by multisystem organ dysfunction appear the most frequent causes of death.

Males and females have been equally affected, with children (0–14 years of age) accounting for 19 %, young adults (15–44 years) 58 %, and older adults (≥45 years) 23 % of reported cases. While the current case fatality proportion in West Africa is approximately 40 %, it has varied substantially over time (highest near the outbreak onset) according to available resources (40–90 % mortality in West Africa compared to under 20 % in Western Europe and the USA), by age (near universal among neonates and high among older adults), and by Ebola viral load at admission.

While there is no Ebola virus-specific therapy proven to be effective in clinical trials, mortality has been dramatically lower among EVD patients managed with supportive intensive care in highly resourced settings, allowing for the avoidance of hypovolemia, correction of electrolyte and metabolic abnormalities, and the provision of oxygen, ventilation, vasopressors, and dialysis when indicated. This experience emphasizes that, in addition to evaluating specific medical treatments, improving the global capacity to provide supportive critical care to patients with EVD may be the greatest opportunity to improve patient outcomes.

**Electronic supplementary material:**

The online version of this article (doi:10.1186/s13054-016-1325-2) contains supplementary material, which is available to authorized users.

## Background

In December 2013, transmission of *Zaire ebolavirus* (Ebola virus (EBOV)) to humans occurred in southeastern Guinea [1], spreading to Liberia and Sierra Leone and rapidly surpassing the cumulative total of previous Ebola virus disease (EVD) outbreaks [2, 3]. Prior outbreaks occurred primarily in remote, resource-challenged settings, with case fatality proportions of 50–88 % [4]. This current outbreak, due to its size and spread in West Africa, in addition to exported and medically evacuated cases to Europe and North America, has engaged a much broader health worker community, including critical care clinicians. While the clinical manifestations, duration of illness, and transmissibility appear similar to previous EVD outbreaks [1, 5–9], with the availability and provision of advanced supportive care in Europe and North America, mortality was less than 20 %, emphasizing the potential importance of supportive and critical care in the management of EVD patients. This review provides an up-to-date examination of EVD using the knowledge gained during the 2013–2016 West African outbreak to highlight relevance for the critical care physician.

## Viral hemorrhagic fevers

Viral hemorrhagic fever describes the syndrome of acute severe febrile illness caused by over 30 viruses from four different taxonomic families—Filoviridae, Arenaviridae, Bunyaviridae, and Flaviviridae. Although they differ in disease epidemiology, transmission, and pathogenesis, most of these RNA viruses are zoonotic and cause nonspecific symptoms including fever, headache, weakness, vomiting, and diarrhea. Infection with filoviruses, including Marburg and Ebola viruses, can be associated with a rapid progression to hemodynamic instability, shock, and multiorgan dysfunction [4, 8].

Filoviruses were discovered in 1967 when 31 laboratory workers became ill after coming into contact with green monkeys imported from Africa [10]. The newly discovered virus killed 23 % of infected workers in the German town of Marburg. Ebola virus was discovered in 1976 during simultaneous outbreaks in Zaire and Sudan in which 88 % and 53 % of patients died, respectively [2, 11, 12]. Since its discovery there have been approximately 25 EVD outbreaks [3].

## Epidemiology

The West African EVD outbreak was first recognized in March 2014 in the forested region of southeastern Guinea. However, the first EVD case may have happened as early as December 2013 with zoonotic transmission of EBOV from an animal to a human, and subsequent human-to-human spread [1]. Transmission was likely well underway throughout West Africa in the spring of 2014 [13]. By June 2014 there were a few hundred confirmed or probable EVD cases, 3000 by the end of August—outstripping the ability of providers at existing Ebola treatment units to isolate and care for patients—and eventually over 20,000 cases by the end of December 2014 [14]. As of 20 May 2016 there have been 28,610 reported confirmed, probable, and suspected EVD cases and 11,308 deaths in Guinea, Sierra Leone and Liberia [15]. Another 36 cases have primarily received care in Mali, Senegal, and Nigeria as well as countries outside Africa including the USA, UK, Germany, Spain, France, Italy, the Netherlands, Norway, and Switzerland (Table 1) (Additional file 1). EVD cases in West Africa have been reported equally among males and females, with children (0–14 years of age) accounting for 19 %, young adults (15–44 years) 58 %, and older adults (≥45 years) 23 % of reported cases [5, 6, 16].

**Table 1:** Chronological demographic description of 27 Ebola virus disease patients treated outside West Africa (August 2014–May 2015)

	Age (years)	Occupation	Country where Ebola virus infection occurred	Case presentation	Country of Hospitalisation	Hospital LOS (days)	Outcome
1^a^	33	Health worker	Liberia	Medically evacuated	USA	19	Survived
2^a^	59	Health worker	Liberia	Medically evacuated	USA	14	Survived
3	75	Non health worker	Liberia	Medically evacuated	Spain	5	Died
4	29	Health worker	Sierra Leone	Medically evacuated	UK	10	Survived
5^a^	36	Health worker	Sierra Leone	Medically evacuated	Germany	30	Survived
6^a^	51	Health worker	Liberia	Medically evacuated	USA	20	Survived
7^a^	43	Health worker	Sierra Leone	Medically evacuated	USA	41	Survived
8	N/A	Health worker	Liberia	Medically evacuated	France	16	Survived
9	70	Nonhealth worker	Sierra Leone	Medically evacuated	Spain	3	Died
10^a^	45	Unknown	Liberia	Imported infection	USA	8	Died
11^a^	38	Health worker	Sierra Leone	Medically evacuated	Germany	47	Survived
12^a^	44	Health worker	Spain	Secondary infection	Spain	30	Survived
13^a^	33	Nonhealth worker	Liberia	Medically evacuated	USA	16	Survived
14	30	Health worker	Sierra Leone	Medically evacuated	Norway	13	Survived
15	56	Health worker	Liberia	Medically evacuated	Germany	6	Died
16^a^	26	Health worker	USA	Secondary infection	USA	13	Survived
17^a^	29	Health worker	USA	Secondary infection	USA	14	Survived
18	33	Health worker	Guinea	Imported infection	USA	19	Survived
19	N/A	Nonhealth worker	Sierra Leone	Medically evacuated	France	21	Survived
20^a^	44	Health worker	Sierra Leone	Medically evacuated	USA	2	Died
21^a^	43	Health worker	Sierra Leone	Medically evacuated	Switzerland	15	Survived
22	50	Health worker	Sierra Leone	Medically evacuated	Italy	38	Survived
23	N/A	Nonhealth worker	Liberia	Medically evacuated	Netherlands	13	Survived
24	39	Health worker	Sierra Leone	Imported infection	UK	25	Survived
25	25	Health worker	Sierra Leone	Medically evacuated	UK	15	Survived
26	N/A	Health worker	Sierra Leone	Medically evacuated	USA	27	Survived
27	N/A	Health worker	Sierra Leone	Imported infection	Italy	31	Survived

^a^Medical management (including utilization of invasive therapies) is described in peer-reviewed format (Table 3) and in reference [40]

*LOS* length of stay (days), *N/A* not available

## Characteristics of transmission

Person-to-person transmission of EBOV occurs through mucous membrane contact with bodily fluids (e.g., vomit, feces, and blood) from those who are infected and symptomatic or by touching the body of someone who died of EVD [17]. Droplet transmission is less likely to occur due to low prevalence of respiratory symptoms [16, 18]. While there are animal transmission models of aerosolized EBOV [19, 20], the clinical relevance of small particle droplet nuclei transmission is unclear and may only apply to the care of critically ill patients undergoing aerosol-generating procedures (intubation and ventilation, bronchoscopy) [18]. Percutaneous transmission with sharps (needle-stick, glass-related exposure) contaminated with infected bodily fluids is thought to be a very efficient mechanism of Ebola virus transmission [11].

## Infection prevention and control practices

**Fig. 1:**
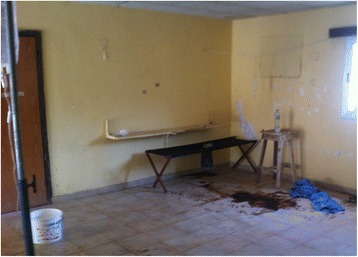
West African Ebola Treatment Facility—April 2014

The concern about EBOV transmission has positioned infection prevention and control (IPC) at the center of clinical care. Theoretically, meticulous provision of contact precautions (hand washing, the use of gloves and a gown along with protection against mucus membrane (eyes, nose, and mouth) exposure, and proper donning and doffing of all personal protective equipment (PPE)) should be sufficient to prevent nosocomial EBOV transmission. In nonclimate-controlled West African Ebola treatment facilities (Fig. 1), the heat and humidity prevent optimal functioning of medical masks and some duckbill N-95 respirators (humidity and sweat cause them to sag and collapse). Tight fitting goggles often fog, reducing visibility. Face shields afford improved visibility but must provide sufficient coverage without risk of splash-related facial exposure. Half-sphere, semi-rigid respirators tend to be more resistant to moisture-related collapse and deformation. However, concern about the high mortality of EVD has led to IPC and PPE choices in the field that sometimes do not follow traditional infection control recommendations or guidance from international technical organizations. This has often resulted in PPE that cannot be safely tolerated by healthcare personnel for more than 45–60 min due to excessive heat and humidity [21], risking syncope, potentially dangerous behaviour (e.g., adjusting fogged facial protection with soiled gloves), and inability to safely perform tasks such as insertion of intravenous catheters. In West Africa, Ebola virus transmission likely also occurs among healthcare personnel during informal healthcare provision in the community to patients or colleagues, without appropriate infection prevention and control practice [22, 23]. Nosocomial EBOV transmission in Spain [24, 25] and the USA [26] similarly reinforces the importance of rigorous Ebola IPC practices and healthcare personnel training, irrespective of the healthcare system [27, 28].

## Pathophysiology

The pathogenesis of EVD in humans remains poorly understood but shows similarities with, and differences from, other causes of viral hemorrhagic fever or bacterial sepsis. End-organ dysfunction seems to result from a combination of a direct viral cytopathic effect, the host immune response, and from under-resuscitated hypovolemic shock [5, 6]. EBOV binds to lectins and other surface receptors, with monocytes, macrophages, and dendritic cells as targets. These virus-containing cells spread through the lymphatic system, liver, and spleen, resulting in a widely disseminated viral infection [29, 30]. Endothelial cell infection and activation may lead to increased levels of soluble adhesion molecules, thombomodulin, and inflammatory mediators such as interferon-gamma and -alpha, interleukins (IL)-2, 6, and 10, interferon-inducible proteins, and tumor necrosis factor alpha [29, 30], resulting in vascular injury.

Thrombocytopenia, consumption, and reduced production of clotting factors, in addition to increased concentrations of fibrin degradation products in patients with severe EVD, may contribute to bleeding [29, 30]. Hepatocellular inflammation is common, and myositis with elevations of creatine kinase and pancreatitis (elevated blood amylase and lipase levels) occurs in severe cases [29, 30].

While acute kidney injury can often be explained by under-resuscitated hypovolemia, it might also arise from viral or secondary bacterial sepsis, acute tubular necrosis, myoglobinuria, and microvascular renal thrombi associated with sepsis or disseminated intravascular coagulation [29, 30]. Adrenal gland viral infection has been shown in animal models and might contribute to hypotension, renal sodium loss, and hypovolemia [30].

## Diagnostics

Diagnostic testing is recommended when a patient exhibits symptoms meeting the EVD case definition [31]. Ebola viral RNA can be detected in clinical specimens by real-time reverse transcription polymerase chain reaction (RT-PCR); if the virus is detected by a specific antigen diagnostic test or by detection of IgM antibodies directed against EBOV, RT-PCR should be used for confirmation [31, 32]. Because the sensitivity of molecular tests depends on Ebola viral loads, specimens collected within 3 days of symptom onset may be falsely negative due to undetectable viremia early in the clinical course. In these circumstances, another blood specimen for RT-PCR testing should be collected 3 days after symptom onset [33]. Point-of-care rapid diagnostic tests (RDTs) have been tested in the field, but they lack sensitivity, require a cold chain, and remain under evaluation in clinical trials [34].

## Clinical presentation of Ebola virus disease

The clinical presentation of EVD falls along a spectrum ranging from minimally symptomatic infection [8] to severe illness with hemorrhagic complications, shock, multiorgan dysfunction, and death. The incubation period ranges from 2 to 21 days but may depend on the mode of transmission [4]: 5–7 days following a percutaneous inoculation and a mean of 9 days following direct mucus membrane contact with infected bodily fluids [8, 30, 35]. EVD typically begins with nonspecific initial signs and symptoms including fever, fatigue, weakness, and headache, similar to many infectious diseases in sub-Saharan Africa, often leading to a missed diagnosis and continued transmission. A fleeting maculopapular rash can be seen within the first week [36, 37]. Gastrointestinal symptoms (nausea, vomiting, abdominal pain, and diarrhea) usually follow 4–6 days after illness onset and can lead to hypovolemia and shock with multisystem organ dysfunction. Gastrointestinal losses and anorexia can precipitate hypokalemia, metabolic acidosis, and acute kidney injury [6]. Hypoxia and ventilation failure tend to occur with severe illness and may be exacerbated by vascular injury and accompanying large-volume fluid requirements [38]. Serious hemorrhagic complications are relatively rare, while more mild bleeding may occur in approximately 30 % of cases [5, 6, 9, 16, 39, 40]. Delirium and encephalopathy or encephalitis may reflect metabolic encephalopathy or direct neuroinvasion and hiccups may be of central or peripheral neurological origin [5, 30, 41, 42].

### Clinical outcomes

While the cumulative case fatality proportion in West Africa is approximately 40 % as of May 2016, it has varied substantially during the course of the outbreak (being higher near the beginning). In comparison, the cumulative case fatality proportion for patients treated in Western Europe and the USA during 2014–2015 was 18.5 % [40] (Table 2). Case fatality remains highest among young children and older adults [5, 6, 16, 43]. Pregnant women often experience spontaneous abortion followed by bleeding, as well as preterm labor and stillbirth if Ebola virus infection occurs later in pregnancy. Vertical transmission and subsequent neonatal mortality has been virtually uniform in the few documented live births by women with acute EVD [44]. A high Ebola viral load at time of admission is associated with more severe illness and mortality [5, 6, 29, 43, 45–47], with other markers of organ dysfunction variably associated with outcomes [6, 8, 29, 48].

**Table 2:** Demographic and outcome summary of 27 Ebola virus disease patients treated outside West Africa

	All	Survived	Died
Treated outside West Africa	27	22 (81.5 %)	5 (18.5 %)
Gender^a^ (male)	17 (68 %)	12 (60 %)	5 (100 %)
Median age^b^ (range)	40.5 (25–75)	36 (25–59)	56 (42–75)
Mean hospital length of stay (days, confidence interval)	19 (±11.5)	22 (±10.2)	5 (±2.4)
Evacuated from West Africa	20 (74 %)	16 (80 %)	4 (20 %)
Infected outside West Africa	3 (11 %)	3 (100 %)	0

^a^Gender available for 25 patients

^b^Age available for 22 patients

### Monitoring and care delivery

The management of critically ill EVD patients in a resource-constrained setting has historically been restricted to variable monitoring of daily clinical signs and symptoms without access to continuous assessment [8]. The need for strict IPC practices and separation of patient care areas in West Africa significantly limited documentation and review of daily clinical records both inside and outside of high-risk patient care. As the case burden decreased and the ratio of healthcare personnel to patients increased, assessments were performed more systematically at some Ebola treatment facilities, with temperature, heart rate, blood pressure, respiratory rate, pulse oximetry, qualitative descriptions of urine, and gastrointestinal output, as well as fluid balance estimation.

Laboratory data other than Ebola RT-PCR results were essentially unavailable at Ebola treatment facilities in West Africa early in the outbreak [33, 49, 50]. Initially, there was very limited attention to diagnostics other than Ebola RT-PCR. The point-of-care systems for monitoring biochemistry and hematology parameters, such as the i-STAT® or the Piccolo Xpress®, were inconsistently utilized inside Ebola treatment facilities [5, 6, 48], in part due to limited manufacturer-recommended temperature and humidity ranges. Over the course of the West African outbreak, with support from national and deployed international laboratories, basic biochemistry, blood counts, and coagulation profiles helped to characterize the course of illness, but remained inconsistently available and often with substantial delays in results reporting due to transport and processing time. Malaria rapid diagnostic tests, and less commonly RT-PCR, were available at most of the laboratories that supported Ebola treatment facilities in West Africa. However, testing for Lassa fever virus (endemic in EBOV-affected countries) or other causes of sepsis was not routine.

Bedside ultrasonography has not been widely deployed [51] but could help with assessing volume status, responsiveness to intravenous fluids [50], and assessment of challenging clinical signs such as abdominal distension [42]. Plain chest and abdomen radiography has been performed in European and North American settings [52] but has rarely been available to patients with Ebola in West Africa. Among patients treated in the USA and Europe, pulmonary edema has been reported in 44 % and acute respiratory distress syndrome in another 22 % [40].

### Discharge criteria and virus persistence during convalescence

The World Health Organization (WHO) recommends considering discharge of patients from isolation on the basis of a negative blood Ebola virus RT-PCR result taken at least 3 days after the resolution of symptoms [33, 41]. However, Ebola virus can persist in certain body fluids after it is undetectable in the blood and after clinical recovery from EVD [42, 53–56]. Viable Ebola virus has been isolated from urine, semen, cerebrospinal fluid, and vitreous humor many months after blood clearance [42, 56–61], suggesting that some activities (unprotected sex, invasive procedures, or penetrating eye trauma) confer a transmission risk even after symptoms and viremia resolve. It is therefore critical to counsel EVD survivors about the risks of Ebola virus persistence [59, 60] and appropriate precautions after discharge, including barrier protection during sexual intercourse [50] until semen has tested negative for Ebola virus twice, or for at least 6 months after EVD onset [62]. Health workers and others should continue to apply standard precautions, as with all patients, when evaluating EVD survivors in the convalescent period. Additional infection prevention and control practices, based upon an individual patient risk assessment, may be prudent for specific procedures in convalescence, even when there is no detectable EBOV in the blood (e.g., lumbar puncture or vitreous humor sampling) [63].

### Critical and supportive care interventions

Providing supportive care to critically ill patients with EVD in resource-poor settings is challenging [64] due to limited infrastructure, lack of materials and trained healthcare personnel, and uncertainty regarding the translation of modern sepsis treatment strategies [65] and optimal intravenous fluid management protocols in the absence of advanced monitoring used in resource-rich settings [66]. Respiratory symptoms such as cough are not a prominent feature of EVD and tachypnea likely represents respiratory compensation of severe metabolic acidosis [9, 30, 50]. Translating fluid resuscitation protocols used in a resource-rich setting [42] to settings where supplemental oxygen therapy is not routinely available [49], in a disease with possible vascular leak syndrome [38], could result in increased morbidity [67] and warrants further investigation [68–70]. The use of antibiotics is common at Ebola treatment facilities [5, 6] before the diagnosis of EVD is confirmed in febrile patients and as empiric treatment of potential bacterial co-infection or gastrointestinal bacterial translocation in patients with confirmed EVD [9]. However, disruption of gastrointestinal flora due to broad-spectrum antibiotics could exacerbate diarrhea and fluid losses. Symptomatic treatment of severe diarrhea with loperamide was variably employed across Ebola treatment facilities. The risks and benefits of these practices warrant evaluation with observational studies and clinical trials [71].

**Fig. 2:**
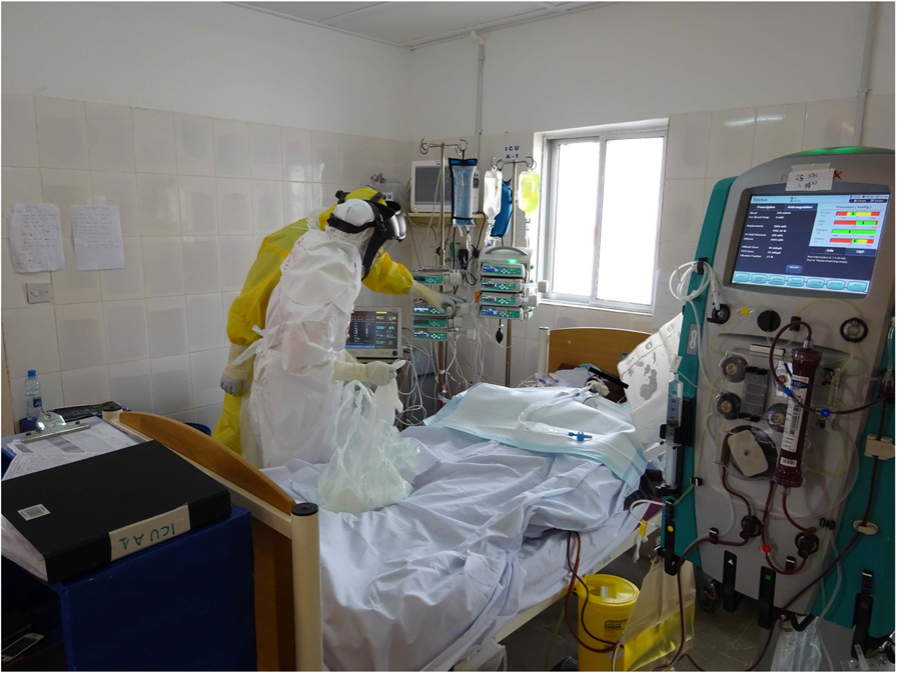
Ebola treatment facility, Goderich, Sierra Leone—February 2015

Early and during the peak of the outbreak, clinical management was generally limited to supportive care focusing on aggressive oral and occasional intravenous volume resuscitation. As the case numbers decreased, advanced care became more common in some treatment facilities. Despite the limitations of working in PPE, feasibility and safety of central venous catheter placement was demonstrated at a UK military-supported treatment facility in Kerry Town, Sierra Leone [72]. Feasibility of transthoracic echocardiography was demonstrated at a military Ebola treatment facility in Conakry, Guinea [51]. By mid-December 2014, EMERGENCY, an Italian nongovernmental organization, established an Ebola critical care unit in Lakka and Goderich, Sierra Leone, the latter consisting of constant bedside nursing, continuous blood pressure, heart rate, respiratory rate monitoring, pulse oximetry, arterial and venous cannulation, nasogastric tube feeding, invasive ventilation, continuous renal replacement therapy, diagnostic biochemistry and hematology, ultrasonography, and plain radiography (Fig. 2). With waning case numbers, accurate evaluation of the impact of these interventions on patient outcomes has not been possible. Other sites, such as the GOAL-supported Mathaska Ebola Treatment Unit (ETU) and the Partners in Health-supported Maforki ETU in Sierra Leona also began using aspects of critical care procedures by February 2015, including nasogastric tube feeding, bedside ultrasound, as well as intraosseus cannulation for intravenous fluid resuscitation [73].

**Fig. 3:**
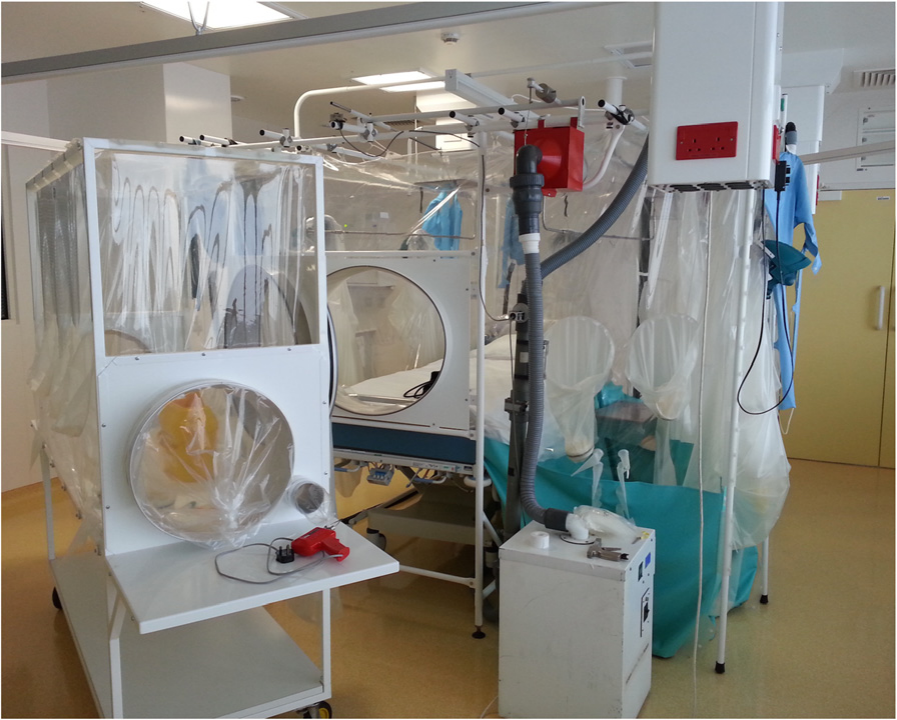
Ebola treatment facility, Royal Free Hospital, London, UK—September 2014

**Fig. 4:**
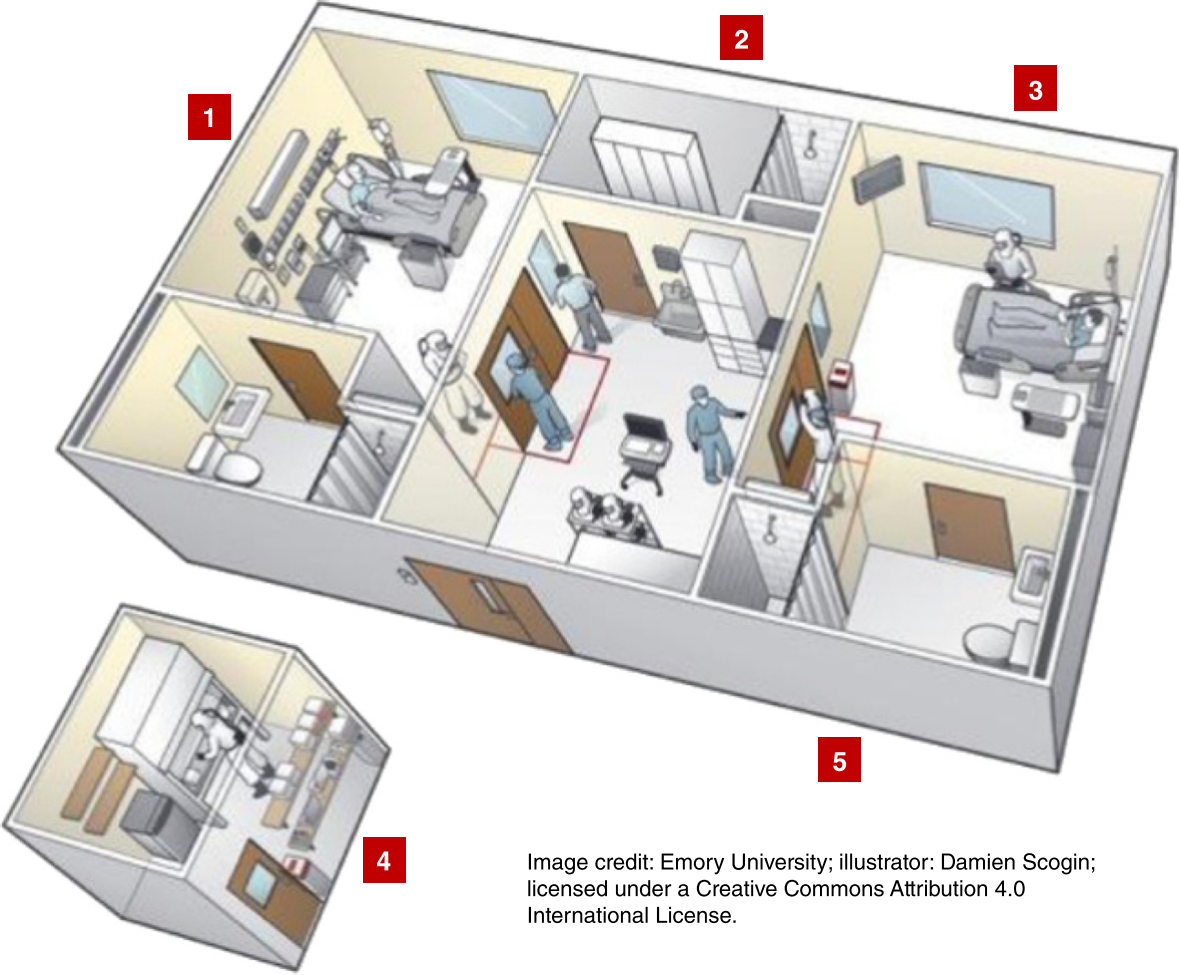
Emory University Hospital special isolation unit. (1) The private patient rooms resemble intensive acre unit rooms, with adjustable beds, intravenous drips, and monitors. Every procedure a patient could need, from mechanical ventilation to hemodialysis, can be performed in the unit. (2) Medical staff who are providing direct patient care use a locker room to change into full-body protective suits and masks, which shield them from blood and bodily fluids. (3) Family members are able to speak with patients through glass windows in the unit; patients have access to phones and laptop computers. (4) A dedicated lab was built specifically for the use with the isolation unit that has the capacity to perform blood counts, routine chemistries, blood gas measurements, urinalysis, and tests for a variety of infectious agents. (5) All liquid waste is disinfected and flushed, and disposable waste is autoclaved and incinerated. At the peak of an Ebola patient’s illness, up to 40 bags a day of medical waste were produced

The historical philosophy of providing only oral fluids for EVD care has given way to the delivery of context-appropriate critical care [38, 42, 74–79]. To date, 27 patients managed in nine countries outside of West Africa (Table 1) have been described, with a survival of 81.5 % [40] (Table 2). Thirteen detailed accounts of EVD management in modern healthcare settings in the USA, Germany, Spain, and Switzerland provide insight into the course of the illness [38, 42, 76–82] (Table 3). These case reports confirm that intensive care monitoring in appropriately prepared centers is feasible. Noninvasive ventilation [38, 42], mechanical ventilation [38, 78, 81], central venous catheter insertion for vasopressor support [38, 42, 78, 79, 82], and renal replacement therapy [38, 78, 81, 82] can be provided effectively and safely (Table 3, Figs. 3 and 4) [83].

**Table 3:** Clinical management summary of 13 Ebola virus disease patients treated outside West Africa

Reference	[76]	[76]	[42]	[28, 79, 137]	[78, 79, 82]	[81, 82]	[38]	[25, 77]	[28, 99]	[81]	[81]	[82]	[37]
Demographics													
Gender	M	F	M	M	M	M	M	F	M	F	F	M	M
Age (years)	33	59	36	51	43	42	38	44	33	26	29	44	43
Country of infection	Liberia	Liberia	Sierra Leone	Liberia	Sierra Leone	Liberia	Sierra Leone	Spain	Liberia	USA	USA	Sierra Leone	Sierra Leone
Country providing care	USA	USA	Germany	USA	USA	USA	Germany	Spain	USA	USA	USA	USA	Switzerland
Hospital admission													
Admission date	2 Aug 2014	5 Aug 2014	27 Aug 2014	5 Sep 2014	9 Sep 2014	30 Sep 2014	3 Oct 2014	6 Oct 2014	6 Oct 2014	11 Oct 2014	14 Oct 2014	15 Nov 2014	21 Nov 2014
Days from diagnosis to evacuation	7	10	4	7	2	N/A	5	0	5	N/A	N/A	6	4
Hospital LOS	19	14	30	20	41	8	47	30	16	13	14	3	15
Vital status	Survived	Survived	Survived	Survived	Survived	Died	Survived	Survived	Survived	Survived	Survived	Died	Survived
Critical care therapies													
Central line	N	N	Y	Y	Y	Y	Y	N	Y	Y	N	Y	Y
Vasopressors	N	N	N	N	Y	Y	Y	N	N	N	N	Y	N
Oxygen	Y	Y	Y	Y	Y	Y	Y	Y	N	N	N	Y	N
NIV (d, days)	N	N	Y (8d)	N	N	N	Y (1d)	N	N	N	N	N	N
MV (d, days)	N	N	N	N	Y (17d)	Y (5d)	Y (13d)	N	N	N	N	Y (3d)	N
CRRT (d, days)	N	N	N	N	Y (24d)	Y (5d)	Y (18d)	N	N	N	N	Y (3d)	N
IHD (d, days)	N	N	N	N	N	N	Y (10d)	N	N	N	N	N	N
Experimental therapies													
Convalescent plasma	Y	N	N	Y	Y	N	N	Y	Y	Y	Y	Y	N
ZMapp/ZMab	Y	Y	N	N	N	N	N	Y	N	Y	N	Y	Y
Brincidofovir	N	N	N	N	N	Y	N	N	Y	Y	Y	N	N
Favipiravir	N	N	Y	N	N	N	Y	Y	N	N	N	N	Y
TKM	N	N	Y	Y	Y	N	N	N	N	Y	N	N	N
Other	N/A	N/A	N/A	N/A	N/A	N/A	Amiodarone, FX06	N/A	N/A	N/A	N/A	N/A	N/A

*CRRT* continuous renal replacement therapy; *F* female, *IHD* intermittent hemodialysis, *LOS* length of stay, *M* male, *MV* invasive mechanical ventilation, *N/A* not available, *NIV* non-invasive ventilation, *TKM* TKM-Ebola, small interfering ribonucleic acids (siRNA) produced by Tekmira

## Establishing supportive and critical care services in highly resourced settings

While it may be advisable to concentrate or regionalize care for patients with EVD in specific hospitals, any health centre should be prepared to safely take a focused and relevant history from a patient with an infectious syndrome, and to mobilize the appropriate local and regional response. Many hospitals, even if not EVD referral centers, may be asked to care for patients until initial (and possibly subsequent) blood Ebola RT-PCR results are known. Therefore, it is essential that hospital staff are well trained and familiar with recommended IPC practices (and for EVD, standard and contact IPC precautions in particular). It is ideal to have an on-call inter-professional team who have undergone training in Ebola-specific IPC training.

While practiced IPC protocols are important to keep health workers safe, a very common clinical pitfall is to equate IPC practices with care. While Ebola-specific standardized IPC protocols are absolutely necessary, there will be situations requiring patient-specific IPC risk-assessments—most commonly involving patients at the beginning of, or in the convalescent phase of, their illness with minimal symptoms and no vomiting or diarrhoea (i.e., with very low risk of transmission). It is also important to remember that most patients suspected with EVD will not have EVD, but will have illness in need of prompt treatment—commonly malaria—that may require empiric treatment while awaiting diagnostic testing [84, 85]. Barriers to providing the standard of care to patients suspected of EVD will repeatedly arise: “We don’t have the capacity to do that… that is not part of our protocol.” Do not accept this when it negatively influences patient care. Instead, ask collectively “How can we safely solve this challenge, now, for the benefit of this patient?”

For hospitals and intensive care units (ICUs) that will provide definitive care for patients with EVD, there are many Ebola-specific considerations well beyond the scope of this review; however, a number deserve mention. Hospitals and ICUs will generally need to mould EVD planning to the local environment, and seek out the experience, guidance, protocols, and training from those who have substantial clinical and operational experience (Figs. 3, 4) [64, 86].

Second, the physical environment of a proposed Ebola treatment unit is a critical component of care. Ideally, there should be a large available physical space, sufficient for multiple isolation rooms, with very generously sized antechamber areas for donning and doffing, and a shared area from where clinical observation can occur (Fig. 4). There should be sufficient adjoining space to house dedicated diagnostic (e.g., portable radiograph and ultrasound machines, potentially point-of-care laboratory devices) and therapeutic (intravenous pumps, mechanical ventilator and circuits, dialysis machine and supplies) equipment. There should be ample nearby space to house packaged soiled PPE and medical waste that allows pick-up and proper disposal.

Third is the necessity for sufficiently numerous and trained inter-professional team of clinical (nurses, physicians, respiratory therapists, others) and patient support staff (coordinators, monitors, cleaners, patient transportation services, diagnostic and laboratory staff, and so forth), who are well practiced in the institutional Ebola care plan and their specific roles. Whether this team is led by infectious disease or critical care specialists, or both, is likely less important than establishing an inter-disciplinary model of continuity care throughout the hospital stay, oftentimes in a single geographic location that is institutionally appropriate.

Fourth, while EVD is accompanied by an increasingly well-characterized clinical gastrointestinal syndrome leading to fluid, electrolyte, and acid-base imbalance with multisystem organ dysfunction, there are no Ebola-specific therapies yet to be proven effective. However, intensive care medicine comprises experiential and evidence-based organ-supportive care, which should guide the care of patients with EVD—attention to fluid, electrolyte and acid-base balance, initiation of empiric and specific anti-infective therapy, and support for renal injury and respiratory failure as occurs for other potentially self-limited and survivable illnesses. Among patients with EVD treated in the USA and Europe, 41 % were deemed to have critical illness with 70 % receiving supplemental oxygen, 22 % with acute respiratory distress syndrome, 26 % invasive mechanical ventilation, 30 % intravenous vasoactive medications, and 19 % requiring dialysis [40].

For the most severely ill patients, clinical judgment is always necessary to balance risks and benefits of certain resuscitation strategies, including the initiation of cardiopulmonary resuscitation (CPR) [28, 87]. While there is a lack of clinical experience with CPR in EVD patients, it may be a reasonable consideration while correcting reversible abnormalities (i.e., hypoxia, severe electrolyte disturbance, arrhythmias) in settings where the option for advanced life-support exists. The decision to provide CPR should be guided by its medical indication and utility in that context, the ability to provide effective CPR, and the safety of those providing care including safe donning and doffing of PPE, in addition to patient preferences [88, 89].

Fifth, as with all critical illness, medical technical care is only one dimension of our support for patients and their families. Patients with EVD and their families require mechanisms to stay in audio and visual contact throughout the illness—ideally visual contact through transparent barriers or at safe distance, or direct contact with supervised donning and doffing of PPE—in addition to substantial psychosocial support during and after EVD.

### Ebola-specific pharmacological prevention and therapeutics

Current EVD treatment focuses on supportive care [70] as there are no specific treatment options yet to be proven effective [70, 90, 91]. A number of Ebola-specific treatment strategies have undergone preliminary clinical trial investigation, including convalescent plasma, Favipiravir, Brincidofovir and TMK-130803 [92–97]. Transfusion of convalescent whole blood or plasma donated by EVD survivors has been used in this and prior EVD outbreaks [98] in an uncontrolled or compassionate-use basis [25, 79, 81, 99], and in animal models [100, 101]. One of three clinical trials of convalescent plasma therapy [94] has been completed and reported [102]. In this nonrandomized comparison to historical controls, transfusion of up to 500 ml convalescent plasma with unknown levels of neutralizing antibodies in 84 patients with confirmed EVD was not associated with a significant improvement in survival. While there were no serious adverse reactions in this trial, transfusion-related acute lung injury was described during convalescent plasma therapy in Spain [25]. Favipiravir (Toyama, Japan) [103], a pre-existing influenza virus inhibitor, has been administered for compassionate use outside West Africa [37, 38, 42]. In a multicenter, nonrandomized clinical trial in Guinea [104], 111 patients receiving Favipiravir had similar survival to that based upon historical control patients. The trial authors suggested that Favipiravir should be further studied in patients with medium to high viremia, but not in those with very high viremia [105]. Brincidofovir (Chimerix, USA), a nucleotide analog that inhibits RNA-polymerase with in vitro activity against Ebola [106], was administered to a small number of EVD patients for uncontrolled compassionate use [42, 79, 81, 99] and was tested in a phase 2 clinical trial in Liberia [95] that was stopped after the manufacturer withdrew study support [107]. TKM-130803 is a formulation of lipid-nanoparticle-encapsulated small interfering ribonucleic acids (siRNA) targeting two proteins involved in Ebola virus transcription and replication (Tekmira, USA, Canada). It was used in nonhuman primate Ebola virus infection as a postexposure treatment strategy [108] and in patients medically evacuated from West Africa in uncontrolled compassionate use [79, 81]. However, a phase 2 clinical trial (RAPIDE-TKM) in Sierra Leone [96] was halted according to pre-established stopping rules [109].

ZMapp, a monoclonal antibody cocktail (Leafbio, USA) [110], has been used under the emergency investigational new drug approvals from the Food and Drug Administration in patients treated in the USA, West Africa, and Western Europe [40, 76, 111]. ZMapp treatment of rhesus macaques resulted in 100 % survival even when started 5 days after lethal EBOV infection [110]. In the only randomized controlled trial of an investigational therapeutic for EVD, ZMapp plus standard of care was compared to standard of care alone for EVD patients in four countries, including the three most impacted West African countries. Due to the decline in EVD cases, this unblinded ZMapp randomized controlled trial only enrolled 72 of the prespecified target goal of 200 EVD patients; data were analyzed for 71 EVD patients, and mortality in the ZMapp treatment group was 22 % versus 37 % in the untreated group, but this difference was not statistically significant [112, 113].

The open-label, uncontrolled, and selected administration of other agents such as amiodarone [114], HMG-CoA reductase inhibitors, and angiotensin II receptor antagonists [115], and therapies to counteract vascular leak (FX06) [38] preclude any conclusions. In an observational study examining temporal trends in mortality among patients with EVD in one ETU in Guinea, 125 of 194 (64.4 %) patients receiving artemether–lumefantrine for malaria prophylaxis died as compared with 36 of 71 patients receiving artesunate–amodiaquine (50.7 %). In adjusted analyses, the risk ratio was 0.69 (95 % confidence interval, 0.54 to 0.89), with a stronger effect observed among patients without malaria [116]. These findings have not been confirmed in a randomized clinical trial.

Two vaccine candidates demonstrated efficacy in nonhuman primates [92, 117, 118]. A recombinant, replication-competent vesicular stomatitis virus-based vaccine expressing a surface glycoprotein of Zaire *ebolavirus* rVSVΔG-EBOV-GP (rVSV) [118, 119] was evaluated in an open-label, ring vaccination trial involving 7651 people in 90 clusters, randomized to immediate or delayed (21 days) administration. The vaccine was well tolerated and in the immediate vaccination group there were no new EVD cases while in the delayed vaccination group there were 16 EVD cases [120]. Another candidate vaccine, cAd3-EBOV (cAd3) [117] remains under investigation [92, 121]. Other vaccine candidates are also under development and evaluation [122, 123].

### Post-exposure Prophylaxis

Several healthcare personnel received post-exposure prophylaxis with different interventions, including a candidate Ebola vaccine, following potential high-risk exposures to Ebola virus; although Ebola virus disease did not occur in these individuals, no conclusions can be made about the effectiveness of these uncontrolled interventions [124–126].

## Ethical challenges in caring for patients with Ebola virus disease

Each of the commonly applied four principles of medical bioethics faces numerous threats in treating patients with EVD [87]. A symptomatic patient’s autonomy to not seek treatment (and not be isolated) is weighed against the threat of disease transmission by staying in the community. The injustice of treatment variability, across regions and over time, places patients at differential risk of death. In acting beneficently, healthcare workers inherently place themselves at some risk. A natural response is to balance that risk with the duty to help. This frequently conspires against greater numbers of health workers responding to an Ebola outbreak. The duty to nonmaleficence, doing no harm, is a daily conundrum, through potential delays in routine diagnostic work-up for common illnesses because of a lack of diagnostic testing, or, in resource-constrained environments, inadequate space to separate potentially infectious suspect patients along a gradient of risk.

## Post-Ebola syndrome

With over 11,000 EVD survivors, there is increased recognition of a post-Ebola syndrome in the convalescent period, characterized by mental health and cognitive sequelae, chronic headaches, insomnia, arthralgias, auditory disturbances, and ocular effects including sight-threatening uveitis [127–132]. It is uncertain whether these manifestations are due to direct viral cytopathic effect in immune-privileged compartments or postinfectious immune-mediated inflammation [133–135].

## Research directions

Although this EVD outbreak narrowed some knowledge gaps, pathophysiology and the immunological response to acute infection and convalescence is still minimally characterized. Access to rapid point-of-care EVD diagnostic capacity to differentiate between other common febrile illnesses [136] is critical because the early presentation of EVD has a broad differential diagnosis [5, 7, 34]. Laboratory testing to identify prognostic indicators could help guide clinical care. Evaluation of specific antiviral therapies is critical as is evaluation of commonly used treatments for which there is still very limited evidence (e.g*.*, empiric antibiotics, anti-diarrheal agents, and fluid replacement composition and volume). The safety and functionality of PPE must be improved. Standardized, easy-to-use clinical charting and human resources for data entry should be made available to support cohort studies and clinical trials. While it seems intuitive that provision of advanced supportive and critical care improves patient outcomes, operationalizing and evaluating increased levels of care to resource-challenged environments is needed. Prepared research protocols that can be rapidly adapted to country-specific settings and quickly implemented could reduce research delays in future outbreaks. Following patients who survive EVD is important to better characterize immune correlates of virus clearance and host genetic factors that contribute to survival, and to better address morbidity of the post-Ebola syndrome.

## Conclusions

The provision of advanced supportive and critical care for EVD patients, while challenging, is possible in both West African and more developed healthcare settings. Creating and evaluating context-appropriate intensive care capacity is a knowledge translation priority. The experience of this outbreak emphasizes that, in addition to evaluating specific medical treatments, improving the global capacity to provide supportive critical care to patients with severe illness may be associated with the greatest opportunity to improve patient outcomes.

## Abbreviations

CPR, cardiopulmonary resuscitation; EBOV, Ebola virus; ETU, Ebola Treatment Unit; EVD, Ebola virus disease; ICU, intensive care unit; IL, interleukin; IPC, infection prevention and control; PPE, personal protective equipment; RT-PCR, real-time reverse transcription polymerase chain reaction.

## Additional file

Additional file 1: Weblink references for Tables 1–3. (DOCX 124 kb)
