# Supplementary material for: Ebola virus disease and critical illness
Source: Crit Care. 2016 Jul 29;20:217. doi: 10.1186/s13054-016-1325-2 (PMC4965892; doi:10.1186/s13054-016-1325-2)
Supplement: Additional file 1: — Weblink references for Tables 1–3. (DOCX 124 kb) [file 13054_2016_1325_MOESM1_ESM.docx]

**References for Tables 1-3**

[1]

[2]

[3]

[4]

[5]

[6]

[7]

[8]

[9]

[10]

[11]

[12]

[13]

[14]

[15]

[16]

[17]

[18]

[19]

[20]

[21]

[22]

[23]

[24]

[25]

[26]

[27]

**References**

1. **Ebola patients discharged from Emory University Hospital** [<http://news.emory.edu/stories/2014/08/er_ebola_press_coverage/campus.html>]

2. Lyon GM, Mehta AK, Varkey JB, Brantly K, Plyler L, McElroy AK, Kraft CS, Towner JS, Spiropoulou C, Stroher U *et al*: **Clinical care of two patients with Ebola virus disease in the United States**. *The New England journal of medicine* 2014, **371**(25):2402-2409.

3. **Ebola: Spanish missionary dies of disease after being flown to Madrid** [<http://www.theguardian.com/world/2014/aug/12/ebola-spanish-missionary-dies-madrid-liberia>]

4. **Ebola: Infected British healthcare worker named as William Pooley** [<http://www.telegraph.co.uk/news/worldnews/ebola/11054634/Ebola-Infected-British-healthcare-worker-William-begged-to-treat-dying-patients.html>]

5. **Ebola patient treated at Hamburg hospital** [<http://www.thelocal.de/20140827/ebola-patient-expected-in-hamburg-who-africa>]

6. **American Ebola patient released from Nebraska hospital** [<http://www.cnn.com/2014/09/25/health/ebola-american-patient/>]

7. **Anonymous Ebola patient released from Emory after being declared virus-free** [<http://www.washingtonpost.com/news/to-your-health/wp/2014/10/20/anonymous-ebola-patient-released-from-emory-after-being-declared-virus-free/>]

8. **French Ebola case to receive test treatment, minister says** [<http://www.reuters.com/article/2014/09/19/us-health-ebola-france-idUSKBN0HE0OH20140919>]

9. **Spanish priest with Ebola in serious condition** [<http://uk.reuters.com/article/2014/09/22/uk-health-ebola-spain-idUKKCN0HH1JZ20140922>]

10. **Details of Duncan’s Treatment Reveal a Wobbly First Response to Ebola** [<http://www.nytimes.com/interactive/2014/10/25/us/ebola-dallas-timeline.html>]

11. **Doctor hospitalized for Ebola in Germany** [<http://www.thelocal.de/20141003/ugandan-doctor-hospitalized-for-ebola-in-germany>]

12. **Ebola crisis: Nurse in Spain tests positive in first infection outside west Africa** [<http://www.theguardian.com/world/2014/oct/06/nurse-spain-tests-positive-ebola>]

13. **American journalist Ashoka Mukpo returns home after recovery from Ebola** [<http://www.theguardian.com/world/2014/oct/23/ashoka-mukpo-ebola-american-released-healthy-hospital>]

14. **Ebola virus victim arrives in Norway by special jet** [<http://www.thelocal.no/20141006/ebola-virus-victim-arrives-in-norway>]

15. **Liberian Ebola patient arrives in Leipzig** [<http://www.thelocal.de/20141008/leipzig-clinic-to-treat-liberia-ebola-doctor>]

16. **Texas nurse who had worn protective gear tests positive for Ebola** [<http://www.cnn.com/2014/10/12/health/ebola/>]

17. **With New Ebola Case Confirmed, U.S. Vows Vigilance** [<http://www.nytimes.com/2014/10/16/us/ebola-outbreak-texas.html>]

18. **Doctor in New York City Is Sick With Ebola** [<http://www.nytimes.com/2014/10/24/nyregion/craig-spencer-is-tested-for-ebola-virus-at-bellevue-hospital-in-new-york-city.html>]

19. **Ebola UN Ebola patient being treated in French hospital** [<http://www.theguardian.com/world/2014/nov/02/ebola-united-nations-worker-france>]

20. **Doctor Being Treated for Ebola in Omaha Dies** [<http://www.nytimes.com/2014/11/18/us/martin-salia-omaha-ebola-dead.html>]

21. **Ebola-infected Cuban doctor arrives in Switzerland for treatment** [<http://www.theglobeandmail.com/news/world/ebola-infected-cuban-doctor-arrives-in-switzerland-for-treatment/article21690324/>]

22. **Ebola outbreak: Doctor becomes first Italian to catch Ebola** [<http://www.bbc.com/news/world-africa-30180345>]

23. **U.N. peacekeeper with Ebola arrives in Netherlands for treatment** [<http://www.reuters.com/article/2014/12/06/us-health-ebola-netherlands-idUSKBN0JK0NC20141206>]

24. **Ebola Patient Is Moved to London, and 2 Others Are Tested in Britain** [<http://www.nytimes.com/2014/12/31/world/europe/ebola-virus-britain.html>]

25. **British medic declared free of Ebola** [<http://www.bbc.com/news/health-32088310>]

26. **Patient admitted with Ebola virus disease discharged from NIH Clinical Center** [<https://www.nih.gov/news-events/news-releases/patient-admitted-ebola-virus-disease-discharged-nih-clinical-center>]

27. **Italian nurse back from Sierra Leone tests positive for Ebola** [<http://www.reuters.com/article/us-italy-ebola-idUSKBN0NX25B20150512>]
